# Structure and Base Analysis of Receptive Field Neural Networks in a Character Recognition Task

**DOI:** 10.3390/s22249743

**Published:** 2022-12-12

**Authors:** Jozef Goga, Radoslav Vargic, Jarmila Pavlovicova, Slavomir Kajan, Milos Oravec

**Affiliations:** Faculty of Electrical Engineering and Information Technology, Slovak University of Technology, Ilkovicova 3, 812 19 Bratislava, Slovakia

**Keywords:** structured receptive fields, shallow neural networks, reproducibility of neural networks, multiscale analysis, fixed kernels

## Abstract

This paper explores extensions and restrictions of shallow convolutional neural networks with fixed kernels trained with a limited number of training samples. We extend the work recently done in research on Receptive Field Neural Networks (RFNN) and show their behaviour using different bases and step-by-step changes within the network architecture. To ensure the reproducibility of the results, we simplified the baseline RFNN architecture to a single-layer CNN network and introduced a deterministic methodology for RFNN training and evaluation. This methodology enabled us to evaluate the significance of changes using the (recently widely used in neural networks) Bayesian comparison. The results indicate that a change in the base may have less of an effect on the results than re-training using another seed. We show that the simplified network with tested bases has similar performance to the chosen baseline RFNN architecture. The data also show the positive impact of energy normalization of used filters, which improves the classification accuracy, even when using randomly initialized filters.

## 1. Introduction

Convolutional neural networks bring, along with considerable advantages, a number of challenging problems. One of them is the choice of hyperparameters for the individual layers. From previous research, we know the effects of the activation function, depth of the network, different types of normalization, preprocessing, and optimization methods on the overall accuracy achieved [1]. The open problem is still the choice of convolutional kernel sizes and their numbers in the individual layers. The popular answer is a minimum symmetric kernel of size 3 × 3 pixels [2], which also contains a small number of parameters. However, this solution is memory-intensive in terms of calculating convolution with high resolution input images, and, therefore, the convolutional kernel can be enlarged with respect to physical constraints [3]. Another reason for changing the kernel size is that it directly affects the size of the receptive field, as the larger filter takes into account the larger surroundings and captures higher-level image features. However, enlarging the size of a kernel quadratically increases the number of its possible parameters and may not improve network accuracy due to generalization problems [4,5]. Moreover, rapid reduction of dimensionality by convolution with a larger kernel leads to shallower architectures. The practical solution is the use of a grid search [6], as there is no general solution yet, and the architectural design of these networks requires some experience.

Another not less-important problem of classic deep networks is their representative complexity, where it is not entirely clear what transformation the network performs and what the individual features mean. Therefore, some authors also believe the direction that research should take is to create simpler rather than deeper models [7,8]. This trend of architecture modifications towards parameter reduction and simplification has become an active area of research in recent years. In this case, it is important to maintain the ability of networks to produce representative features with an emphasis on achieved accuracy.

Last but not least, the need for a large amount of data for the training process itself significantly limits the wider deployment of deep learning. Although there are ways to achieve good results on smaller datasets, such as using transfer learning and expanding the training set by image augmentation [9], these techniques should be implemented with caution. The use of transfer learning is, in many cases, application-specific [10], and image augmentation techniques can create small image artefacts that must be considered in critical applications such as medical diagnostics [11].

### 1.1. Simplification of Convolutional Neural Networks

With regard to the success of deep neural networks, the main motivations for their simplification are deployment on devices with limited computing resources, as well as a deeper understanding and easier interpretation of the created models. One of the major simplifications was tested by Iandola et al. [12], who proposed the SqeezeNet architecture. In addition to the introduction of Fire Modules that reduce the dimensionality of data in the first step, followed by an expansion phase, the same authors also introduced complex bypass connections. These bridging connections are similar to those in the ResNet [13] architecture but with an introduction of the point-wise convolution operation. The overall architecture has 50-fold fewer parameters while retaining similar accuracy as the AlexNet [3] architecture. Another point of interest is that the fully connected layers were not used in the Sqeezenet architecture intended for classification. Instead of the common approach, one feature map for each corresponding category of the classification task was generated. On top of these feature maps, the average of each feature map was taken separately, resulting in an output vector that was then directly fed into the softmax layer. This approach is generally called global average pooling and was introduced by Lin et al. [14] when proposing Network in Network architecture. On the success of previous architectures, Howard et al. [15] presented an efficient convolutional neural network designed for deployment on mobile devices based on depthwise separable convolution [16].

One of the other approaches is the replacement of some convolutional kernels by approximated Gabor filters. In convolutional computational layers, these filters are used as fixed kernels for the extraction of intrinsic features. Some authors used Gabor filters only in the first convolutional layer [17,18], while Sarwar et al. [19] also looked at their use in other layers with an increasing depth of the network. Adding fixed filters to deeper layers of the network, however, results in a decrease in overall accuracy. Therefore, the same authors proposed a solution that uses a combination of fixed and trainable kernels. The interim results show that this method leads to a significant improvement in energy savings, shortened training time, and reduced memory requirements with a negligible drop in classification accuracy. The combination of learned free-form filters with structured ones also bring the ability to modify the size of convolutional kernels in the training process [20]. It can lead to an effective expansion of the total number of convolutional kernels [21] or reduction of parameters by creating efficient layers for feature extraction [22,23].

In addition to these methods, there are many approaches based on compression, regularization, and pruning [24,25].

### 1.2. Path towards Receptive Field Neural Networks

One of the possible solutions that could at least partially solve these problems is to replace standard convolutional kernels with a linear combination of predefined and fixed filters in convolutional neural network architectures. This influential concept, proposed by Jacobsen et al. [26], defines Receptive Field Neural Networks (RFNNs) as a group of networks that do not handle convolutional kernels as individual pixels but as functions in scale-space using a finite Gaussian derivative basis. The architecture of RFNNs allows the use of any unspecified base, which creates efficient filters designed for feature extraction. It is therefore not surprising that models based on fixed filters according to the original Receptive Field Neural Network (RFNN) architecture have aroused the interest of researchers in recent years. Schlimbach [27] showed that filter size has an impact on network performance, and it was therefore proposed to include scale as a parameter in the learning process itself. This idea was later elaborated by Pintea et al. [28], who created a network that effectively learns the dynamic size of the convolutional kernel. A similar approach was developed by Tabernik et al. [22,23], who created a Displaced Aggregation Units (DAUs) capable of learning the receptive field size through spatial displacement. By extending the original model with the Gaussian base of multiple scales and orientations, the authors of [29] created a Multi Scale and Orientation Structured Receptive Neural Network (MSO-RFNN) inspired by the DenseNet architecture [30].

Although the architecture of RFNNs are specific to their Gaussian derivative basis, other bases have also been tested. Verkes [31] proposed using the Gabor kernels, where real and imaginary parts are rotated to match a reference Gaussian basis accordingly, and achieved similar results. Discrete directional Parseval frames with compact support were tested in the role of a base by Labate et al. [32]. With the introduction of sparsity constraints, the Geometric-Biased CNN (GBCNN) was created and is capable of solving the problem of hyperspectral image classification. By incorporating Parseval frames into RFNN Resnet-like models, the ResnetRF architecture was proposed by Karantzas et al. [33]. Ulicny et al. [34] proposed using spectral filters based on a Discrete Cosine Transform (DCT), thus creating harmonic neural networks achieving fine accuracy in classification and segmentation tasks.

Although several bases have been proposed (Table 1), a valid comparison is still lacking, especially in the area in which these models excel: training with a small number of training samples. This implies our intention to verify the possibility of using different bases in the RFNN architecture under deterministic conditions.

In this work, we analyse and extend RFNN to study the consequences of changes in the individual compact bases. We restrict RFNN into shallow architectures to look more closely at the impact of different parameters and introduce a deterministic methodology for obtaining reproducible results and evaluation in the form of a Correlated Bayesian *t*-test.

The paper is structured as follows. In Section 2, we review in detail the RFN layer, which is the crucial layer for the RFNN architecture. In Section 3, we present the reference architecture chosen as the baseline for the experiments. The methodological changes that enable reproducibility of experiments are proposed in Section 4. The performed experiments and their results are presented in Section 5. The obtained results are discussed in Section 6. We conclude our contribution in Section 7.

## 2. Receptive Field Neural Layer

In neural network architectures, a convolution layer is commonly used for feature extraction. It may differ in implementation, but usually this layer performs a discrete convolution denoted by the ∗ operator between the input tensor $h^{x}\in R^{1\times D_{x}\times W_{x}\times H_{x}}$ and the set of convolutional kernels $K\in R^{N_{K}\times D_{x}\times W_{K}\times H_{K}}$, which, at the same time represent the set of parameters of this computational layer. The output is a multidimensional feature map $h^{y}\in R^{1\times N_{K}\times \left(W_{x}-W_{K}+1\right)\times \left(H_{x}-H_{K}+1\right)}$, which is created by applying convolution kernels in all mutual positions to the input where the kernel fits entirely within the input boundaries. Equation (1) shows the calculation of the *j*-th feature map $h_{j}^{y}\in R^{1\times 1\times \left(W_{x}-W_{K}+1\right)\times \left(H_{x}-H_{K}+1\right)},j\in \left\{0,1,2,\ldots ,N_{K}-1\right\}$ in a standard 2D convolutional layer, where $q\in \{0,1,2,\ldots ,W_{x}-W_{K}\}$ and $p\in \{0,1,2,\ldots ,H_{x}-H_{K}\}$ are their horizontal and vertical indices, respectively.

(1) $$\begin{array}{cc} h_{j}^{y}\left(p,q\right) & =2DConv\left(h^{x},K_{j}\right)\left(p,q\right)=\left(h^{x}\times K_{j}\right)\left(p,q\right)\\ & =\sum_{i=0}^{D_{x}-1}\sum_{u=0}^{H_{K}-1}\sum_{v=0}^{W_{K}-1}h^{x}\left(i,p+u,q+v\right)\cdot K_{j}\left(i,H_{K}-u-1,W_{K}-v-1\right) \end{array}$$

We can decompose the regular convolution into two simpler operations: extraction of features in the spatial plane using dephtwise convolution and subsequent channel recombination using pointwise convolution, effectively creating a depthwise separable convolutional layer [44,45]. Depthwise convolution is convolution in the spatial domain applied independently over every channel of an input. The calculation of the feature maps ${\tilde{h}}^{y}\in R^{1\times N_{K}D_{x}\times \left(W_{x}-W_{K}+1\right)\times \left(H_{x}-H_{K}+1\right)}$ by the 2D depthwise convolution is described by Equation (2), where $i\in \{0,1,2,\ldots ,D_{x}-1\}$ and $k\in \{0,1,2,\ldots ,N_{K}-1\}$. The output is a three-dimensional tensor, where the index $o\in \{0,1,2,\ldots ,N_{K}D_{x}-1\}$ represents a specific channel in the depth plane, and $p\in \{0,1,2,\ldots ,H_{x}-H_{K}\}$ and $q\in \{0,1,2,\ldots ,W_{x}-W_{K}\}$ are their vertical and horizontal indices, respectively.

(2) $$\begin{array}{cc} {\tilde{h}}^{y}\left(o,p,q\right) & =2DDepthwiseConv\left(h^{x},\Theta \right)\left(o,p,q\right)\\ & =\sum_{u=0}^{H_{K}-1}\sum_{v=0}^{W_{K}-1}h^{x}\left(i,p+u,q+v\right)\cdot \Theta \left(k,i,H_{K}-u-1,W_{K}-v-1\right) \end{array}$$

Subsequent pointwise convolution can be seen as regular convolution with a 1×1 kernel size, recombining the channels along depth and projecting them onto a new channel space and creating an output *j*-th feature map $h_{j}^{y},j\in \left\{0,1,2,\ldots ,D_{Y}-1\right\}$ (Equation (3)).

(3) $$\begin{array}{cc} h_{j}^{y}\left(p,q\right)=PointwiseConv\left({\tilde{h}}^{y},\alpha_{j}\right)\left(p,q\right) & =\sum_{o=0}^{N_{K}D_{x}-1}{\tilde{h}}^{y}\left(o,p,q\right)\cdot \alpha_{j}\left(o\right) \end{array}$$

The original set of parameters K is hereby replaced by $\Theta \in R^{N_{K}\times D_{x}\times W_{K}\times H_{K}}$ as a set of effective filters and $\alpha \in R^{D_{Y}\times N_{K}D_{x}\times 1\times 1}$ for the subsequent recombination of the created feature maps.

The main idea of the RFNN computational layer is to replace the parameters of 2D spatial filters Θ with $N_{K}$ fixed-base filters θ. This makes it possible to introduce information into the network a priori in the form of fixed spatial 2D convolutional filters for the extraction of intrinsic features, which are then linearly combined to create effective feature maps $h^{y}$ at the output of the RFNN layer (Equation (4)).

(4) $$\begin{array}{c} h^{y}=PointwiseConv\left(2DDepthwiseConv\left(h^{x},\theta \right),\alpha \right)\; \end{array}$$

**Figure 1:**
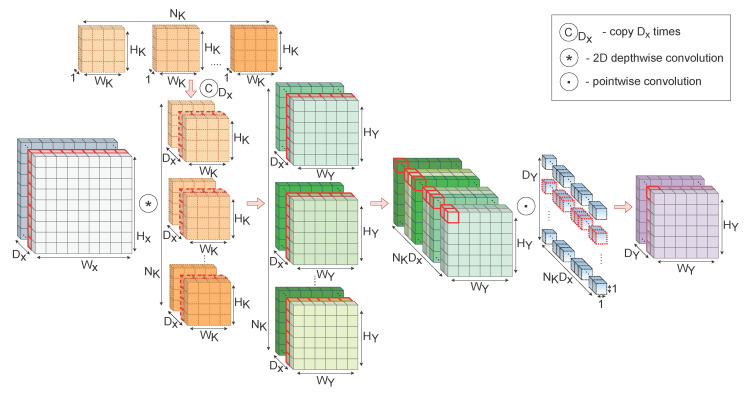
Receptive Field Neural Layer. The set of $N_{K}$ fixed filters is first copied $D_{x}$ times to equal the number of input channels. Subsequently, intrinsic features are extracted using the depthwise convolution, and the output feature maps are created in the learning process by a linear combination using a pointwise convolution.

By introducing fixed filters from a certain domain, the convolutional kernels can be treated as functions, while the network parameters are also reduced, as only α parameters are learned during the training process. The detailed architecture of the RFNN layer is shown in Figure 1.

The hyperparameters of this layer are:

- A set of fixed two-dimensional kernels ($N_{K}$—number of kernels; $W_{K}$, $H_{K}$—width and height of kernels);
- The convolution parameters used for feature extraction (padding, stride, and possibly other additional parameters);
- The number of output feature maps ($D_{Y}$—depth dimension of the output tensor).

## 3. Choice of Reference Architecture

As a baseline for comparative evaluation of RFNN networks, we selected the methodology and results from Jacobsen et al. [26], as described below. We re-implemented their proposed RFNN network to reproduce the originally published results and to ensure correct implementation using the PyTorch [46] framework. We refer to this implementation as $RFNN_{ref}$. Since the original article contains several experiments and architectural modifications, we decided to concentrate on results where the MNIST [47] dataset was used (Figure 5 in the original paper [26]).

The baseline architecture is a convolutional neural network with three layers of receptive fields and is used to classify the known problem of handwritten digits into ten classes. A detailed description of the individual layers together with the output dimensions and number of parameters is given in Table 2.

Fixed convolution kernels from the Gaussian basis were formed in the same way for all layers with a difference in size. In the first layer, the convolutional kernels have a size of 11×11 pixels and σ=1.5, while in the second and third layers the kernels are identical with a size of 7×7 pixels with σ=1. In each receptive fields layer, filtered outputs after depthwise convolution are recombined into 64 output feature maps.

## 4. Proposed Changes

Although we managed to verify the performance of the baseline architecture (see Section 5.1) and even achieve better results (see Section 5.2) than the original authors using the baseline architecture, we noticed the shortcomings of such an evaluation, which is common in the field of neural networks. The results may be affected by the setting of hyperparameters and training methodology, randomness of selection and order of samples, as well as stochastic regularization (e.g., dropout) during training. These effects are more pronounced with a smaller training set, which, in our case, was manifested by an increase in variance. We encountered the difficulties when reproducing the original results. Therefore we propose to change to a methodology that ensures reproducible results. This methodology is also used in our experiments, starting in Section 5.3:

- All used frameworks were set to deterministic mode;
- All parameters were initialised based on one chosen experimental seed;
- To ensure variability, the selected experimental seed was used to generate a seed vector for all runs of the experiment.

**Figure 2:**
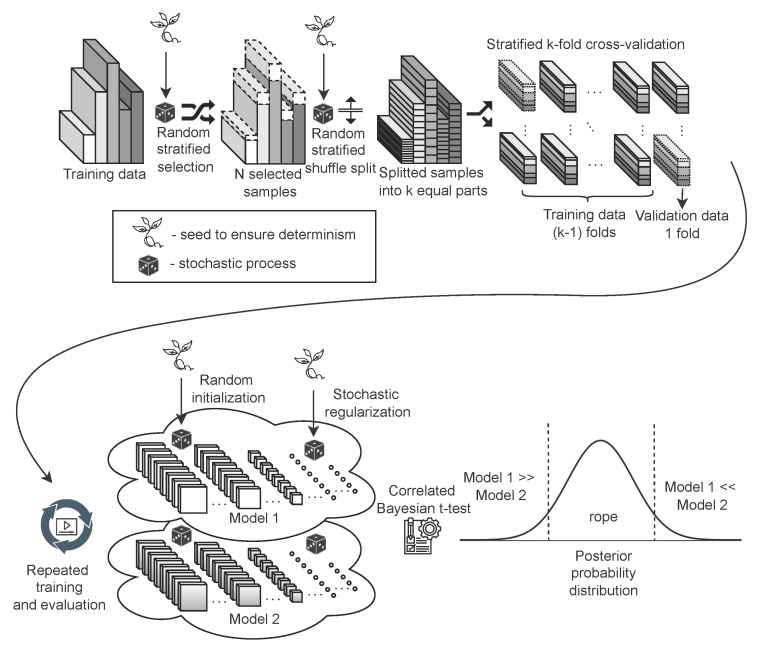
The training and evaluation methodology consisted of stratification and repeated cross-validation while ensuring repeatability by setting a deterministic mode in all parts of the experiment. The final evaluation is realized in the form of a Correlated Bayesian *t*-test.

Based on the recommendations in [48,49] we chose the Bayesian-based comparison in the form of a Correlated Bayesian *t*-test [50]. We used an implementation of this test from the Baycomp library [48]. The results of this test are three probabilities: that the models are practically equivalent, that model C1 achieves better results than model C2, and vice versa. For all tests, we chose the standard 1% as the Region of Practical Equivalence (ROPE), where if the difference in the metrics of the compared models A and B is less than or equal to 1%, we consider the models to be equivalent. Based on previous adjustments and evaluations, we decided to change the methodology of the experiments. Subsequently, in each experiment, all frameworks using randomness were set to the deterministic mode. We used stratified 10-fold cross-validation repeated 10 times with different initial seedings (a total of 100 simulations). The same master seed is used for each experiment, so both the selection and the order of the training samples are maintained for all models on the same dataset. The statistical evaluation of the model in the form of Correlated Bayesian *t*-test was performed on the validation part of the training set within the cross validation. However, a numerical evaluation was performed on the excluded test set containing the same 10,000 samples to obtain an objective view of the real accuracy of the model. A possible alternative is to combine the training and test sets into one dataset. We chose not to use this procedure in order to be able to compare the results obtained on a dedicated test set with previous research in this area. The final training and evaluation methodology for ensuring repeatability is shown in Figure 2.

## 5. Experiments and Results

In this section, we describe our experiments with RFNN architecture using the MNIST database. In Section 5.1, we describe our procedure for repeating the results from the original work [26]. Next, in Section 5.2, we experiment with training methodology by applying only one change at a time (random or stratified sampling, early stopping, or different optimizer) to analyse its influence on the accuracy. To see the impact of changes in the RFNN architecture, we simplified the reference network. This simplification is specified in Section 5.3. Next, in Section 5.4, we analyse the impact of sample selection and energy normalization on the achieved accuracy. In Section 5.5, we experiment with different bases instead of a Gaussian compact base, and last, in Section 5.6, we remove the pooling layer and provide corresponding learning curves.

### 5.1. Experiment 1—Reference Architecture Evaluation

Receptive field neural networks excel when not enough data are available to use common deep learning models. We used a limited number of training samples in the range from 300 to 60,000 samples, i.e., the whole training set. For each experiment, the entire MNIST test set, consisting of 10,000 samples, was used to evaluate the classification accuracy. Apart from normalizing data to the range <0,1>, no other form of sample pre-processing was used. The network was trained by a standard backpropagation algorithm using an average cross-entropy loss with a mini-batch size of 25 images. An Adadelta optimizer with decay rate ρ=0.95, stability constant $\epsilon =1\times 10^{-6}$ and linear learning rate decay was applied. The initial value of the learning rate was set to $lr_{i}=5$ and decreased to the final value of $lr_{f}=0.05$ during the training, while a fixed number of epochs were taken from the original article. The values are listed in Table 3 [26].

**Figure 3:**
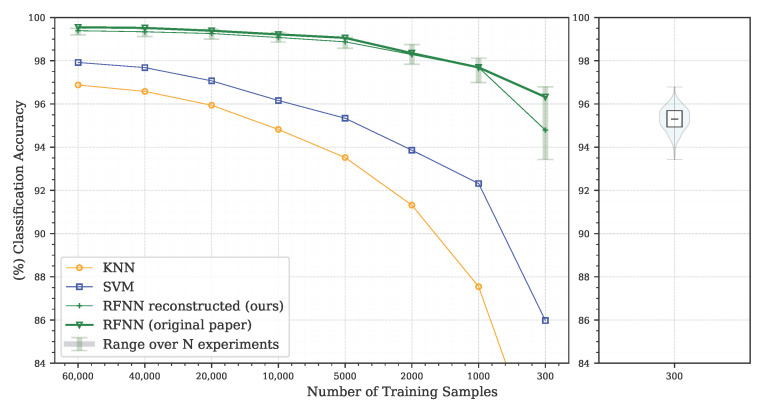
Reproduction of the originally published results on the MNIST database. The average of the three experiments is shown in green for all samples listed in Table 3. Accuracies of the standard KNN and SVM machine learning classifiers are given as separate curves. The graph also shows the range of results obtained for repeating the experiment N = 300 times for the reference architecture $RFNN_{ref}$. For the smallest number of training samples, the estimated distribution of all repeated experimental results shown as a violin plot is also displayed separately (best viewed in colour).

The resulting classification accuracy of the reference architecture $RFNN_{ref}$ representing the average of three experiments is depicted in Figure 3. The original and our result lines approximately match, but there is apparent difference in the last point for the 300 training samples. The variability of these results even if the original methodology is followed may be due to various factors such as the random number generator (RNG) initial state, hardware parameters, software versions of used frameworks and others. These data are missing in the majority of published studies [51], which makes the verification of original results more difficult.

Since we were unable to achieve the original published values for three repetitions, we decided to repeat each experiment 300 times and evaluate the results by displaying the full range <min,max> of the achieved accuracy. The resulting interval is also shown in Figure 3. For reference, we compared the $RFNN_{ref}$ model with standard machine learning classifiers such as k-nearest neighbors (KNN) and support vector machine (SVM). We used an SVM classifier with a radial basis function kernel (RBF), a unit regularization parameter and an automatic kernel coefficient gamma. A KNN classifier with a uniform weight function and 5 neighbors was trained using a brute-force search with standard Euclidean distance as a metric. The range of achieved values is represented in the following part of the article by a violin plot [52]. In addition to the interquartile range, this type of graph also shows the estimated distribution of data. In our representation, we marked the median and displayed the entire range of values from min to max.

We can confirm that we have managed to achieve comparable results to those presented by Jacobsen et al. [26]. The $RFNN_{ref}$ reference architecture achieves a significant improvement in classification accuracy compared to standard machine learning models in our reproduction test on the MNIST database if the number of training samples is limited.

### 5.2. Experiment 2—Reference Architecture and Limited Number of Training Samples

**Figure 4:**
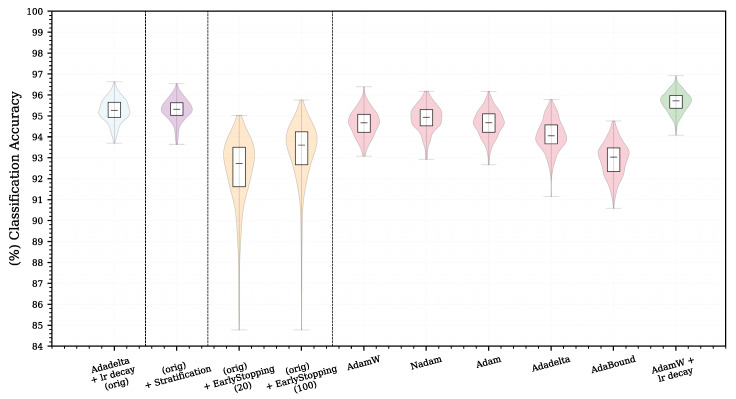
Comparison of various modifications of the methodology on the MNIST database when selecting 300 random training samples (i.e., it corresponds to a value of 300 on the x-axis in Figure 3).

In this section, we focus on the most important contribution of the RFNN architecture, which is the ability to achieve high accuracy with a limited number of training samples. Analysing the results, we observed a high variability of classification accuracy for the smallest number of training samples, which is evident from the violin graph (Figure 3). From these results, it is not possible to determine whether the high variance is due to sampling, the characteristics of the chosen model, or by specific training parameters. To analyse the variance and impact of parameter changes, we tested several adjustments and modifications of the training methodology. Network training and pre-processing were done exactly the same as in the previous section with a random selection of 300 training samples. We repeated the experiment N = 300 times, making only one change in the methodology at a time. The results are shown in Figure 4 and also numerically in Table 4. Note, the leftmost case in Figure 4 is the same as the violin plot (rightmost case) in Figure 3.

#### 5.2.1. Stratified Sampling

First, we replaced random with stratified sampling as a way to eliminate sampling bias and thus a possible source of variance. The resulting violin graph in Figure 4 is denoted as “(orig) + Stratification”. We achieved comparable results to those of the reference methodology, and thus, the possible class imbalance did not have a significant impact on the observed variance.

#### 5.2.2. Early Stopping

In the following experiment, in addition to the original methodology, we used early stopping as follows: 10% of the 300 random samples were selected via stratified sampling in the validation set. The remaining 90% of samples were used for training. During training, the cross-entropy loss on the validation set was evaluated after each epoch. If this loss was reduced, we saved the current parameters of the model and continued training. If the validation loss did not improve during $p=M_{maxepochs}$, then we stopped the training and used the best model whose parameters we had saved. We performed two different experiments for the conditions of early stopping, p=20 and 100, to evaluate the impact of this parameter. When testing a longer stopping condition p=100, better results were obtained, but they had still deteriorated. The results show that a longer stop condition generally produces better results, which is consistent with the results of Prechelt [53]. Figure 4 shows that the use of early stopping can increase the variance of the results, especially if the stop condition is too strict. The deterioration of the results may be affected by the small number of samples in the validation set and by the use of a dropout in the architecture. However, their impact on the overall result would need to be examined in more detail. Due to mixed opinions on the use of early stopping and deteriorating results in our particular case, we decided not to use early stopping in further experiments.

#### 5.2.3. Optimizer Modification

Choi et al. [54] showed that not only does the choice of the optimizer but also the setting of its parameters has a fundamental influence on the model accuracy. However, since tuning the optimizer hyperparameters would require a separate optimization process, and a grid search would be computationally intensive, we decided to test the latest optimizers with the default settings recommended by their respective authors without using the learning rate decay. We used Adadelta [55], Adam [56], AdamW [57], AdaBound [58] and Nadam [59] gradient descent optimization algorithms. The results of our tests (Figure 4 and Table 4) show that the optimizers are almost as successful—only AdaBound and Adadelta lag behind at this setting. In the last comparison, we decided to use the learning rate decay for the most successful optimizer, AdamW, as well as in the original experiments, where we managed to surpass the original results.

**Table 4:** Results of various modifications of the training methodology for analysis of variance. The better results obtained compared to the reference values in the first row are highlighted in bold.

Methodology	Mean Accuracy [%]	Min Accuracy [%]	Max Accuracy [%]	Variance $\left[{\%}^{2}\right]$
Adadelta + lr decay (orig)	95.26	93.70	96.63	0.3033
(orig) + Stratification	**95.30**	93.64	96.54	**0.2684**
(orig) + Early Stopping (20)	92.39	84.78	95.03	2.7026
(orig) + Early Stopping (100)	93.28	84.78	95.76	2.3816
AdamW	94.64	93.08	96.39	0.3854
Nadam	94.88	92.93	96.18	0.3993
Adam	94.65	92.67	96.17	0.3889
Adadelta	94.08	91.15	95.78	0.5439
AdaBound	92.94	90.59	94.76	0.6310
AdamW + lr decay	**95.67**	**94.08**	**96.92**	**0.2204**

### 5.3. Experiment—Simplification of RFNN Architecture

For a valid comparison of the impact of changes in the RFNN architecture, we simplified the reference network as much as possible. Due to the possible mutual influence of parameters in deep architectures, we omitted the normalization and regularization layers and simplified the neural network to a shallow architecture with one computational RFConv layer. The architecture is described in detail in Table 5.

Another simplification was the removal of computational layers (local response normalization and dropout) for which we could not ensure repeatability when training on a GPU. We kept the base and number of filters according to the original architecture. For training, we used the same settings as in the previous experiments except for changing the optimizer to AdamW using a fixed learning rate with a value of $1\times 10^{-3}$. For a reference, we evaluated the simplified 1-layer architecture $RFNN_{L1}$ against the original 3-layer architecture $RFNN_{ref}$ in the form of 10 times repeated 10-fold cross-validation using a Correlated Bayesian *t*-test for the random selection of 300 samples.

**Figure 5:**
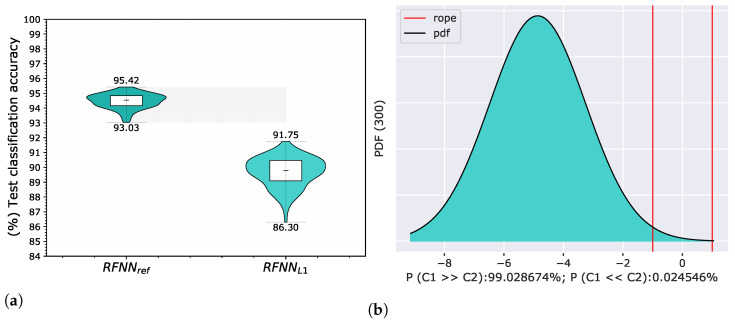
Comparison of $RFNN_{ref}$ and $RFNN_{L1}$ neural model on the MNIST database using proposed methodology for random selection of 300 training samples. The evaluation of the results is shown for test classification accuracy represented by violin graph (**a**) and posterior probability distribution between $RFNN_{ref}$ and $RFNN_{L1}$ of a Correlated Bayesian *t*-test for the validation classification accuracy at 1% ROPE (**b**).

Figure 5 shows the achieved accuracies on a test set in the form of a violin graph and posterior probability distribution of a Correlated Bayesian *t*-test evaluated on a validation set in each cross-validation split. Compared to the original architecture, the results deteriorated significantly, which was expected. For a ROPE of 1%, the probability that $RFNN_{ref}$ was better than $RFNN_{L1}$ was 99.03%, the probability that both models were practically equivalent was 0.95%, and the probability that $RFNN_{L1}$ was better than $RFNN_{ref}$ was 0.02%.

### 5.4. Experiment 4—Sample Selection and Energy Normalization

In this experiment, we analysed the impact of sample selection and Energy Normalization on the achieved accuracy of the created model. The settings and methodology of the training were the same as in the previous section. We chose $RFNN_{L1}$ as the reference architecture.

#### 5.4.1. Sample Selection

**Figure 6:**
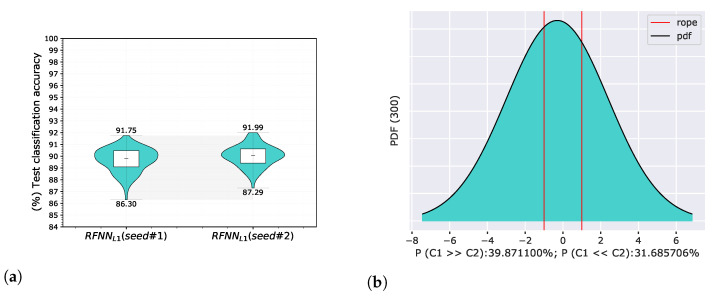
Comparison of $RFNN_{L1}$ neural models trained on different samples based on two deterministic seeds on the MNIST database using proposed methodology. The evaluation of the results is shown for test classification accuracy represented by violin graph (**a**) and posterior probability distribution between $RFNN_{L1}\left(seed\#1\right)$ and $RFNN_{L1}\left(seed\#2\right)$ of a Correlated Bayesian *t*-test for the validation classification accuracy at 1% ROPE (**b**).

We conditioned the selection of samples by changing the deterministic seed that affected the result. We trained again on a random selection of 300 samples and re-evaluated classification accuracies using the same methodology. A comparison of the results obtained with the seed change is shown in Figure 6.

The results differ due to a different selection of samples for the training set despite the same $RFNN_{L1}$ architecture. The probability that the model $RFNN_{L1}\left(seed\#1\right)$ was better than $RFNN_{L1}\left(seed\#2\right)$ was 39.87%, the probability that $RFNN_{L1}\left(seed\#2\right)$ was better than $RFNN_{L1}\left(seed\#1\right)$ was 31.69%, and the probability that both models were practically equivalent was 28.44%=100%−(39.87%+31.69%).

#### 5.4.2. Basis Energy Normalization

**Figure 7:**
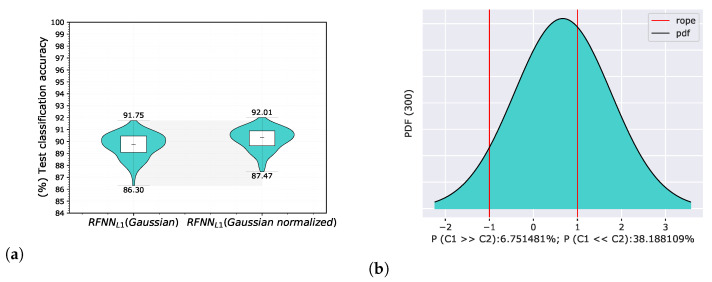
Comparison of $RFNN_{L1}$ neural models with Gaussian derivative basis with and without energy normalization according to Equation (5) on the MNIST database using proposed methodology for random selection of 300 training samples. The evaluation of the results is shown for test classification accuracy represented by violin graph (**a**) and posterior probability distribution between $RFNN_{L1}\left(Gaussian\right)$ and the same model with the energy normalized base $RFNN_{L1}\left(Gaussian\;normalized\right)$ of a Correlated Bayesian *t*-test for the validation classification accuracy at 1% ROPE (**b**).

**Figure 8:**
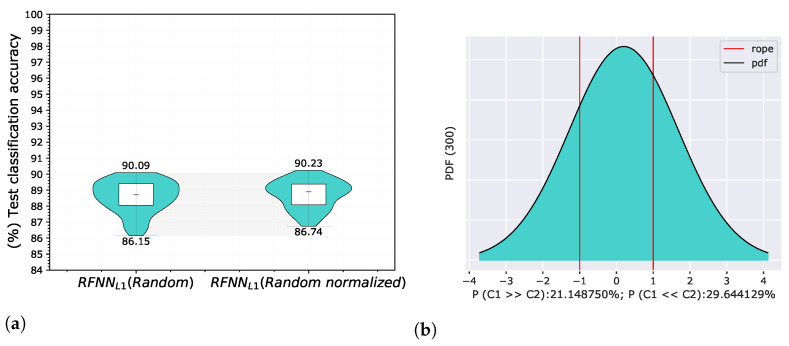
Comparison of $RFNN_{L1}$ neural models with Randomly initialized kernels with normal distribution with and without energy normalization according to Equation (5) on the MNIST database using proposed methodology for random selection of 300 training samples. The evaluation of the results is shown for test classification accuracy represented by violin graph (**a**) and posterior probability distribution between $RFNN_{L1}\left(Random\right)$ and the same model with the energy normalized base $RFNN_{L1}\left(Random\;normalized\right)$ of a Correlated Bayesian *t*-test for the validation classification accuracy at 1% ROPE (**b**).

Normalization in neural network architectures can help to improve both convergence and generalization [60]. Different types of preprocessing and normalization are introduced to eliminate sharp differences between network parameters or extracted feature maps [61]. This effect can be even more pronounced when using fixed filters. For this reason, we evaluated the impact of energy normalization on the achieved accuracy. By energy normalization, we mean normalization to unit energy, where the sum of all squared *k*-th filter elements $\theta_{k}\in R^{W_{K}\times H_{K}}$ is equal to one (Equation (5)).

(5) $$\begin{array}{c} \theta_{k}^{norm}\left(m,n\right)=\frac{\theta_{k}\left(m,n\right)}{\sqrt{\sum_{u=0}^{H_{K}-1}\sum_{v=0}^{W_{K}-1}\theta_{k}^{2}\left(u,v\right)}} \end{array}$$

where $m\in \{0,1,2,\ldots ,H_{K}-1\}$ and $n\in \{0,1,2,\ldots ,W_{K}-1\}$ are their corresponding vertical and horizontal indices. The achieved accuracies on a test set in the form of a violin graph and posterior probability distribution of a Correlated Bayesian *t*-test evaluated on a validation set in each cross-validation split are shown in Figure 7 for the Gaussian derivative basis, and in Figure 8 for randomly initialized kernels.

The results of both experiments show that base energy normalization helped to improve classification accuracy. When using the original Gaussian derivative base, we found that $RFNN_{L1}\left(Gaussian\right)$ was better than $RFNN_{L1}\left(Gaussian\;normalized\right)$ with a probability of 6.75%, the probability that both models were practically equivalent was 55.06%, and the probability that $RFNN_{L1}\left(Gaussian\;normalized\right)$ was better than $RFNN_{L1}\left(Gaussian\right)$ was 38.19%. In the case of using a random base, the probability that $RFNN_{L1}\left(Random\right)$ was better than $RFNN_{L1}\left(Random\;normalized\right)$ was 21.15%, the probability that both models were practically equivalent was 49.21%, and the probability that $RFNN_{L1}\left(Random\;normalized\right)$ was better than $RFNN_{L1}\left(Random\right)$ was 29.64%.

### 5.5. Experiment 5—Basis-Related Experiments

**Figure 9:**
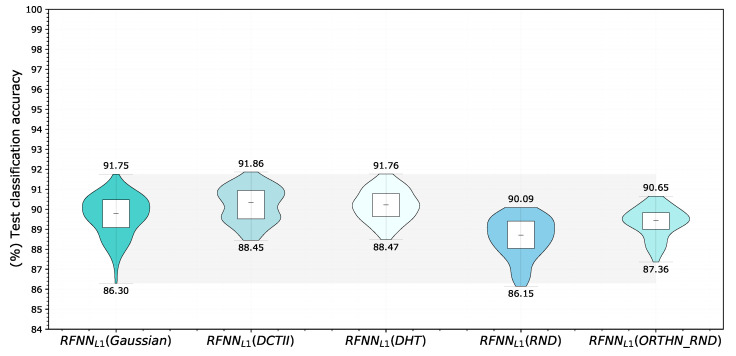
Comparison of different bases of simplified $RFNN_{L1}$ architecture. Each violin graph represents test classification accuracy of $RFNN_{L1}$ neural model with different compact basis on the MNIST database. The test was performed for a random selection of 300 training samples using proposed methodology.

In the original article [26], the Gaussian compact base was used for feature extraction. The aim of our experiment was to verify whether we can replace the given base and what effect this change will have on the achieved classification accuracy. We again chose $RFNN_{L1}$ as the reference architecture, while the parameters and evaluation methodology remained unchanged. In each experiment, we kept all parameters constant except for the base used, which had a size of 11 × 11 pixels. Since the reference Gaussian base contained 10 filters, we selected these filters along the primary diagonal based on a triangular selection according to Ulicny et al. [34]. We compared the reference Gaussian derivative base (Gaussian), the orthonormal Discrete Cosine base (DCTII), the orthonormal Discrete Hartley base (DHT), the randomly initialized kernels with normal distribution (RND), and the random orthonormal base (ORTHN_RND). The results for the selection of 300 samples are shown in Figure 9.

**Figure 10:**
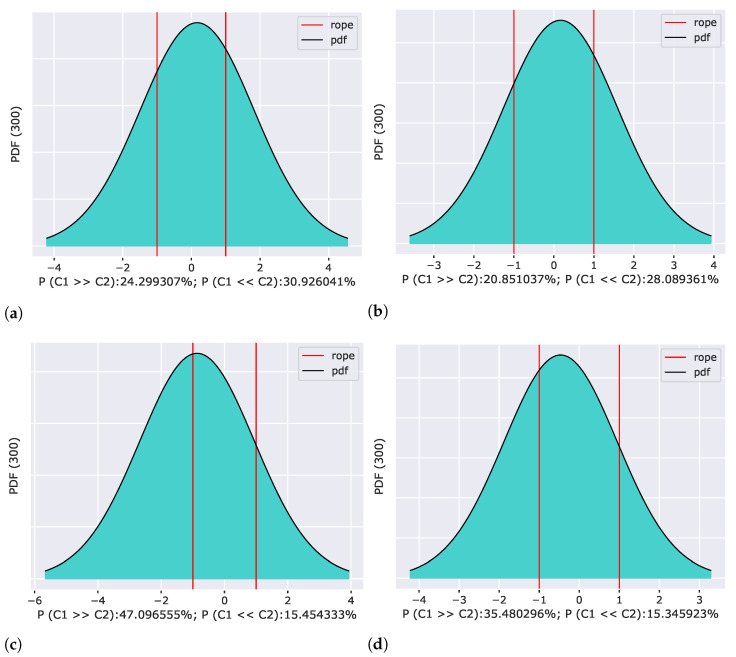
Posterior probability distributions of a Correlated Bayesian *t*-test between reference simplified $RFNN_{L1}$ with Gaussian basis and (**a**) orthonormal discrete cosine basis (DCTII), (**b**) orthonormal discrete Hartley basis (DHT), (**c**) randomly initialized kernels with normal distribution (RND), and (**d**) orthonormal random basis (ORTH_RND). The vertical lines define a region of practical equivalence where the mean difference in accuracy is within ±1%. The test was performed for a random selection of 300 training samples using the proposed methodology.

We evaluated neural models using a Correlated Bayesian *t*-test with respect to the simplified reference architecture $RFNN_{L1}$ with a Gaussian base for each model change separately (Figure 10). The results showed that for all tested bases, the hypothesis that both compared models are equivalent has the highest probability, with the one exception being a random base, which worsened the results and had the greatest impact on achieved classification accuracy. The probability that the $RFNN_{L1}$ model of a network with a Gaussian base is better than a model with a random kernels is about 47.1%, which far exceeds the other results. Compared to the other bases tested, we obtained the best results using discrete Hartley and discrete cosine bases. In both cases, the results of the statistical test were that the models were practically equivalent, with a probability of 51.06% and 44.77%, respectively.

### 5.6. Experiment 6—Learning Curve Analysis and Max-Pooling Removal

**Figure 11:**
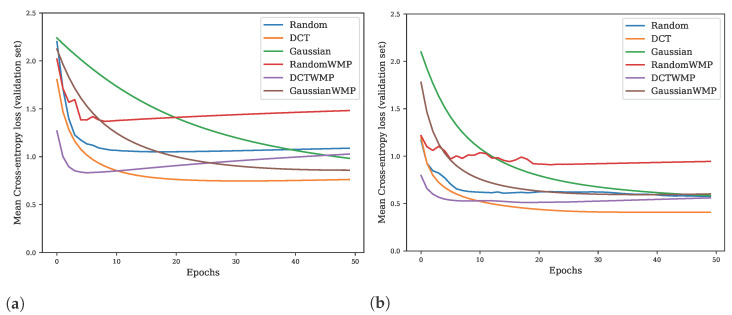
Learning curves of $RFNN_{L1}$ neural models with and without max-pooling (WMP) layer displayed for Randomly initialized base, DCT, and Gaussian derivative basis on the Kuzushiji-MNIST database using proposed methodology for random selection of 300 training samples (**a**) and 1000 training samples, respectively (**b**).

In the previous experiment in Section 5.5, we analysed the influence of the type of fixed basis used. As we can see in Figure 9 and Figure 10, the random kernels also performed relatively well, which could be due to the simplicity of the MNIST database used, or excessive downsampling in max-pooling layer. Based on the obtained results, we decided to explore the influence of the max-pooling layer on the classification accuracy. We compared the network performance with and without the max-pooling layer on the more complex Kuzushiji-MNIST [62] database. The $RFNN_{L1}$ model was trained for a fixed number of 50 epochs using the AdamW optimizer with default setting while following the same methodology and preprocessing as in the previous experiments. We show the corresponding representative results in the form of the learning curves in Figure 11. It can be seen from the results, that the difference between randomly initialized filters and structured bases widens significantly when max-pooling is omitted.

## 6. Discussion

The outcomes of this paper have provided insight into the Receptive Field Neural Network architecture, which is specific in that it uses a linear combination of a predefined fixed kernels to create a set of effective filters instead of learning entire convolutional kernels pixel-by-pixel. The results in Section 5.1 confirm the previously published results on the MNIST database (Figure 3) by Jacobsen et al. [26] and demonstrate the possibility of replacing the original Gaussian derivative base with other structured bases intended for the extraction of intrinsic features within the shallow RFNN architecture. Since the RFNN excels compared to other architectures when training with a limited amount of data; the experiments focused on the random selection of N=300 training samples only while maintaining repeatability of results by introducing a deterministic evaluation methodology (Figure 2). The methodology change was introduced to ensure reliability of findings with respect to the high variability of the achieved classification accuracy on the test set (Section 5.1 and Section 5.2), which we could not reduce by the introduction of sample stratification, the use of early stopping during training, or by changing the optimizer (Figure 4 and Table 4), as shown in Section 5.2. We modified the training methodology to ensure repeatability and evaluated the proposed step-by-step changes in the form of a Correlated Bayesian *t*-test using 10 times repeated 10-fold cross-validation. By switching to this evaluation methodology, we ensured a reproducible environment for reliable comparison of results where not only the selection but also the order of the samples during training remained the same. To ensure repeatability within the neural network, we simplified the original $RFNN_{ref}$ (Table 2) architecture to a single-layer network $RFNN_{L1}$ (Table 5) with removal of normalization and regularization layers for which we could not guarantee determinism within the used framework. At the cost of a significant deterioration of $RFNN_{L1}$ classification accuracy compared to the baseline (Section 5.3, Figure 5), we obtained reproducible conditions for further experiments (Section 5.4 and Section 5.5). When evaluating the influence of the seed for the pseudorandom number generator, on which depends samples selection and the initialization of parameters within the $RFNN_{L1}$ network in the form of a Correlated Bayesion *t*-test, we found that the probability that the same model with different initial seedings and training sets are practically equivalent was only 28.44% (Figure 6b). This probability was the lowest among all experiments, meaning that this change had the greatest impact on the achieved classification accuracy. The results are in line with the findings of Crane et al. [51], who pointed out that unreported effects in evaluation methodology can substantially influence the achieved results. The data also show the positive impact of energy normalization (Section 5.4), which improved the classification accuracy not only for the Gaussian derivative base (Figure 7) but also for randomly initialized fixed filters (Figure 8). The next experiment was a comparison of specifically structured bases within the $RFNN_{L1}$ architecture (Section 5.5). Although we managed to outperform the original Gaussian derivative basis using DCT and DHT bases (Figure 9), the more important result is that the Bayesian Correlated *t*-test (Figure 10) did not show a significant difference between individual bases in terms of achieved classification accuracy with the exception of randomly initialized filters (Table 6). In the last experiment (Section 5.6, Figure 11), we tested the impact of the max-pooling layer. The results show that omitting max-pooling increased the difference between structured bases and randomly initialized kernels. Further research could be devoted to initialization with structured filters based on various transforms, which could, in theory, improve convergence, as shown recently by Li et al. [63]. Since the feature extraction in our reference RFNN architecture is divided into filtering using a set of filters along the spatial dimensions and the subsequent combination of channels via pointwise convolution, representation of the extracted features and the subsequent visual display can be easier to interpret compared to the classical convolutional layers. Therefore, it would be interesting to examine the visual properties of these architectures with respect to the extraction of intrinsic features for better understanding and deployment in areas with a lack of training data, in which they excel.

## 7. Conclusions, Limitations, and Future Research Work

Although these results confirm the added value of structured filters within the RFNN architecture, the differences between the specific bases chosen in the context of neural networks need further investigation, mainly in the context of the various constraints introduced. Deployments of complex neural networks in critical applications and devices with limited computing power are the main reasons for simplification and deeper analysis of created models. In this work, we analysed the Receptive Field Neural Network (RFNN) as a promising simplified model that uses a Gaussian derivative basis inspired by the scale-space theory. When validating the original results, we identified a problem with a large variation in the achieved test classification accuracy, especially with a small amount of training data. To enable the reproducibility of the results by introducing deterministic methodology, we simplified the baseline RFNN architecture to a single-layer CNN network whose input of computational layers was a set of fixed filters of any size and number. Subsequently, we experimentally verified that bases other than Gaussian can be used as fixed filters within the RFNN architecture. We found that a change in base may have less of an effect on the results obtained than re-training the network using another seed in failing to ensure repeatability of results. We also verified the positive impact of energy normalization of used filters, which improves the achieved classification accuracy even when using randomly initialized kernels. Further research is needed to establish specific differences between individual bases and the influence of various hyperparameters within the RFNN architecture.

## Figures and Tables

**Table 1:** A comparison of proposed fixed kernels from a predefined dictionary and their applications within different convolutional neural network architectures inspired by the Receptive Field Neural Network in recent years.

Authors	Proposed Network	Reference Architecture	Fixed Kernels
Jacobsen et al. (2016) [26]	Receptive Field Neural Network	multi-layer CNN, Network in Network	Gaussian derivative basis $\left(11\times 11,7\times 7\right)$
Verkes (2017) [31]	Gabor RFNN	multi-layer CNN	Gabor kernel family $\left(7\times 7\right)$
Schlimbach (2018) [27]	Gaussian RFNN	multi-layer CNN	Gaussian derivative basis $\left(9\times 9,7\times 7,5\times 5,3\times 3\right)$
Hilbert et al. (2018) [29]	Multi-Scale and -Orientation RFNN	DenseNet	Gaussian derivative basis $\left(11\times 11,5\times 5\right)$
Karantzas et al. (2019) [33]	ResnetRF	ResNet18, ResNet34, ResNet50	directional Parseval frames with compact support $\left(7\times 7,3\times 3\right)$
Labate et al. (2019) [32]	Geometric-Biased CNN	multi-layer CNN	directional Parseval frames with compact support $\left(5\times 5,3\times 3\right)$
Ulicny et al. (2019, 2019, 2022) [34,35,36]	Harmonic Network	multi-layer CNN, Wide residual network, SE-ResNeXt, Resnet-50, Resnet-101	DCT basis (various)
Kumawat et al. (2020) [37]	Depthwise-STFT Based Separable Convolutional Neural Network	MobileNet, ShuffleNet, ReLPU	Short-Term Fourier Transform kernels $\left(5\times 5,3\times 3\right)$
Pintea et al. (2021) [28]	N-JetNet	Network in Network, ALLCNN, Resnet-32, Resnet-110, EfficientNet	Gaussian derivative basis (dynamic size)
Tomen et al. (2021) [38]	Deep Continuous Network	ResNet-34, ODE-Net	Gaussian derivative basis (dynamic size)
Saldanha et al. (2021) [39]	FracSRF Network	Network in Network, Resnet-32, EfficientNet-b0	Gaussian derivative basis (dynamic size)
Lindeberg (2021, 2022) [40,41]	Scale-covariant and Scale-invariant Gaussian Derivative Network	multi-layer CNN	scale-normalized Gaussian derivative basis (various)
Fukuzaki et al. (2022) [42]	OtX	VGG-16, DenseNet-121, EfficientNetV2-S, NFNet- F0	Principal Component Analysis of well-trained filter weights $\left(3\times 3\right)$
Penaud–Polge et al. (2022) [43]	Fully Trainable Gaussian Derivative Network	VGG-16, U-Net	oriented and shifted Gaussian derivative kernels (various)

**Table 2:** Baseline reference neural network architecture ($RFNN_{ref}$) with 3 receptive field convolutional layers used in the classification task on the MNIST dataset. The output dimension of individual computational layers is denoted in the form N@H×W×D, where N is the number of objects with height, width and depth, accordingly.

Layer Number and Type	Inner Computational Layers	Hyperparameters	Output Shape	Learnable Parameters
#0 Input	-	-	N@28×28×1	0
#1 RFConv	Depthwise convolution	fixed Gaussian kernels of shape 10@11×11×1, stride = 1, padding = 5	N@28×28×10	0
	Pointwise convolution	number of kernels = 64, stride = 1, padding = 0	N@28×28×64	640
	Max pooling	kernel size = 3×3, stride = 2	N@13×13×64	0
	ReLu	-	N@13×13×64	0
	Local response normalization	size = 9, $\alpha =1\times 10^{-4}$, β=0.75, k=2.0	N@13×13×64	0
	Dropout	*p* = 0.2	N@13×13×64	0
#2 RFConv	Depthwise convolution	fixed Gaussian kernels of shape 6@7×7×64, stride = 1, padding = 6	N@19×19×384	0
	Pointwise convolution	number of kernels = 64, stride = 1, padding = 0	N@19×19×64	24,576
	Max pooling	kernel size = 3×3, stride = 2	N@9×9×64	0
	ReLu	-	N@9×9×64	0
	Local response normalization	size = 9, $\alpha =1\times 10^{-4}$, β=0.75, k=2.0	N@9×9×64	0
	Dropout	*p* = 0.2	N@9×9×64	0
#3 RFConv	Depthwise convolution	fixed Gaussian kernels of shape 6@7×7×64, stride = 1, padding = 6	N@15×15×384	0
	Pointwise convolution	number of kernels = 64, stride = 1, padding = 0	N@15×15×64	24,576
	Max pooling	kernel size = 3×3, stride = 2	N@7×7×64	0
	ReLu	-	N@7×7×64	0
	Local response normalization	size = 9, $\alpha =1\times 10^{-4}$, β=0.75, k=2.0	N@7×7×64	0
	Dropout	*p* = 0.7	N@7×7×64	0
#4 Flatten	Reshape	-	N@3136	0
#5 Linear	Fully-connected	-	N@10	31,360
#6 Output	Softmax	-	N@10	0

**Table 3:** Determined number of epochs of individual experiments for a limited number of samples on the MNIST database.

Number of samples	60,000	40,000	20,000	10,000	5000	2000	1000	300
Number of epochs	100	100	100	100	150	200	300	1000

**Table 5:** Simplified neural network architecture $RFNN_{L1}$ with one receptive field convolutional layer.

Layer Number and Type	Inner Computational Layers	Hyperparameters	Output Shape	Learnable Parameters
#0 Input	-	-	N@28×28×1	0
#1 RFConv	Depthwise convolution	fixed Gaussian kernels of shape 10@11×11×1, stride = 1, padding = same	N@28×28×10	0
	Pointwise convolution	number of kernels = 64, stride = 1, padding = 0	N@28×28×64	640
	Max pooling	kernel size = 3×3, stride = 2	N@13×13×64	0
	ReLu	-	N@13×13×64	0
#4 Flatten	Reshape	-	N@10,816	0
#5 Linear	Fully connected	-	N@10	108,160
#6 Output	Softmax	-	N@10	0

**Table 6:** Results of test classification accuracies on MNIST database obtained using simplified $RFNN_{L1}$ architecture with various fixed bases of the same size for 10 selected filters. The best results are highlighted in bold.

Base	Mean Accuracy [%]	Min Accuracy [%]	Max Accuracy [%]	Variance $\left[{\%}^{2}\right]$
Gaussian	89.67	86.30	91.75	1.0131
Discrete Cosine (DCTII)	**90.21**	88.45	**91.86**	0.7268
Discrete Hartley (DHT)	90.20	**88.47**	91.76	0.5958
Random (RND)	88.48	86.15	90.09	0.9813
Orthonormal random (ORTHN_RND)	89.32	87.36	90.65	**0.5528**

## Data Availability

The experiments in this paper were performed on the public “KMNIST Dataset” [62] (created by CODH), adapted from the “Kuzushiji Dataset” (created by NIJL and others): http://doi.org/10.20676/00000341 and MNIST [47] datasets.

## References

[1] Mishkin D., Sergievskiy N., Matas J. (2017). Systematic Evaluation of Convolution Neural Network Advances on the Imagenet. Comput. Vis. Image Underst..

[2] Garcia-Garcia A., Orts-Escolano S., Oprea S., Villena-Martinez V., Garcia-Rodriguez J. (2017). A Review on Deep Learning Techniques Applied to Semantic Segmentation. arXiv.

[3] Krizhevsky A., Sutskever I., Hinton G.E. (2017). ImageNet Classification with Deep Convolutional Neural Networks. Commun. ACM.

[4] Ozturk S., Ozkaya U., Akdemir B., Seyfi L. Convolution Kernel Size Effect on Convolutional Neural Network in Histopathological Image Processing Applications. Proceedings of the 2018 International Symposium on Fundamentals of Electrical Engineering, ISFEE 2018.

[5] Karatzoglou A., Schnell N., Beigl M. (2020). Applying Depthwise Separable and Multi-Channel Convolutional Neural Networks of Varied Kernel Size on Semantic Trajectories. Neural Comput. Appl..

[6] Chollet F. (2017). Deep Learning with Python.

[7] Yao H., Chuyi L., Dan H., Weiyu Y. Gabor Feature Based Convolutional Neural Network for Object Recognition in Natural Scene. Proceedings of the Proceedings—2016 3rd International Conference on Information Science and Control Engineering, ICISCE 2016.

[8] Rudin C. (2019). Stop Explaining Black Box Machine Learning Models for High Stakes Decisions and Use Interpretable Models Instead. Nat. Mach. Intell..

[9] Shorten C., Khoshgoftaar T.M. (2019). A Survey on Image Data Augmentation for Deep Learning. J. Big Data.

[10] Weiss K., Khoshgoftaar T.M., Wang D.D. (2016). A Survey of Transfer Learning. J. Big Data.

[11] Hussain Z., Gimenez F., Yi D., Rubin D. (2017). Differential Data Augmentation Techniques for Medical Imaging Classification Tasks. AMIA Annu. Symp. Proc..

[12] Iandola F.N., Han S., Moskewicz M.W., Ashraf K., Dally W.J., Keutzer K. (2016). SqueezeNet: AlexNet-level accuracy with 50x fewer parameters and <0.5MB model size. arXiv.

[13] He K., Zhang X., Ren S., Sun J. Deep Residual Learning for Image Recognition. Proceedings of the IEEE Computer Society Conference on Computer Vision and Pattern Recognition.

[14] Lin M., Chen Q., Yan S. Network in Network. Proceedings of the 2nd International Conference on Learning Representations, ICLR 2014.

[15] Howard A.G., Zhu M., Chen B., Kalenichenko D., Wang W., Weyand T., Andreetto M., Adam H. (2017). MobileNets. arXiv.

[16] Sifre L., Mallat S. Rotation, Scaling and Deformation Invariant Scattering for Texture Discrimination. Proceedings of the IEEE Computer Society Conference on Computer Vision and Pattern Recognition.

[17] Chang S.Y., Morgan N. Robust CNN-Based Speech Recognition with Gabor Filter Kernels. Proceedings of the Annual Conference of the International Speech Communication Association.

[18] Li J., Wang T., Zhou Y., Wang Z., Snoussi H. Using Gabor Filter in 3D Convolutional Neural Networks for Human Action Recognition. Proceedings of the Chinese Control Conference.

[19] Sarwar S.S., Panda P., Roy K. Gabor Filter Assisted Energy Efficient Fast Learning Convolutional Neural Networks. Proceedings of the International Symposium on Low Power Electronics and Design.

[20] Shelhamer E., Wang D., Darrell T. Efficient Receptive Field Learning by Dynamic Gaussian Structure. Proceedings of the ICLR 2019 Workshop.

[21] Luan S., Chen C., Zhang B., Han J., Liu J. (2018). Gabor Convolutional Networks. IEEE Trans. Image Process..

[22] Tabernik D., Kristan M., Leonardis A. Spatially-Adaptive Filter Units for Deep Neural Networks. Proceedings of the IEEE Computer Society Conference on Computer Vision and Pattern Recognition.

[23] Tabernik D., Kristan M., Leonardis A. (2020). Spatially-Adaptive Filter Units for Compact and Efficient Deep Neural Networks. Int. J. Comput. Vis..

[24] Li J.Y., Zhao Y.K., Xue Z.E., Cai Z., Li Q. (2019). A Survey of Model Compression for Deep Neural Networks. Gongcheng Kexue Xuebao/Chinese J. Eng..

[25] Blalock D., Gonzalez Ortiz J.J., Frankle J., Guttag J. What is the state of neural network pruning? In Proceedings of the 3rd MLSys Conference, Austin, TX, USA, 2–4 March 2020.

[26] Jacobsen J.H., Van Gemert J., Lou Z., Smeulders A.W.M. Structured Receptive Fields in CNNs. Proceedings of the IEEE Computer Society Conference on Computer Vision and Pattern Recognition.

[27] Schlimbach R.J. (2018). Investigating Scale in Receptive Fields Neural Networks. Bachelor’s Thesis.

[28] Pintea S.L., Tomen N., Goes S.F., Loog M., Van Gemert J.C. (2021). Resolution Learning in Deep Convolutional Networks Using Scale-Space Theory. IEEE Trans. Image Process..

[29] Hilbert A., Veeling B.S., Marquering H.A. Data-Efficient Convolutional Neural Networks for Treatment Decision Support in Acute Ischemic Stroke 2018. Proceedings of the 1st Conference on Medical Imaging with Deep Learning (MIDL 2018).

[30] Huang G., Liu Z., Van Der Maaten L., Weinberger K.Q. Densely Connected Convolutional Networks. Proceedings of the Proceedings—30th IEEE Conference on Computer Vision and Pattern Recognition, CVPR 2017.

[31] Verkes G. (2017). Receptive Fields Neural Networks Using the Gabor Kernel Family. Bachelor’s Thesis.

[32] Labate D., Safaripoorfatide M., Karantzas N., Prasad S., Foroozandeh Shahraki F. Structured Receptive Field Networks and Applications to Hyperspectral Image Classification. Proceedings of the Wavelets Sparsity XVIII.

[33] Karantzas N., Safari K., Haque M., Sarmadi S., Papadakis M. Compactly Supported Frame Wavelets and Applications in Convolutional Neural Networks. Proceedings of the Wavelets Sparsity XVIII.

[34] Ulicny M., Krylov V.A., Dahyot R. (2022). Harmonic Convolutional Networks Based on Discrete Cosine Transform. Pattern Recognit. Pattern Recognit..

[35] Ulicny M., Krylov V.A., Dahyot R. Harmonic Networks for Image Classification. Proceedings of the British Machine Vision Conference (BMVC).

[36] Ulicny M., Krylov V.A., Dahyot R. Harmonic Networks with Limited Training Samples; In Proceedings of the 27th European Signal Processing Conference, EUSIPCO, Coruña, Spain, 2–6 September 2019.

[37] Kumawat S., Raman S. Depthwise-STFT Based Separable Convolutional Neural Networks. Proceedings of the 2020 IEEE International Conference on Acoustics, Speech and Signal Processing (ICASSP).

[38] Tomen N., Pintea S.-L., Gemert J. Van Deep Continuous Networks. Proceedings of the 38th International Conference on Machine Learning.

[39] Saldanha N., Pintea S.L., Van Gemert J.C., Tomen N. (2021). Frequency Learning for Structured CNN Filters with Gaussian Fractional Derivatives. arXiv.

[40] Lindeberg T. (2022). Scale-Covariant and Scale-Invariant Gaussian Derivative Networks. J. Math. Imaging Vis..

[41] Elmoataz A., Fadili J., Quéau Y., Rabin J., Simon L. (2021). Scale Space and Variational Methods in Computer Vision: 8th International Conference, SSVM 2021, Virtual Event, May 16–20, 2021, Proceedings.

[42] Fukuzaki S., Ikehara M. (2022). Principal Components of Neural Convolution Filters. IEEE Access.

[43] Penaud–Polge V., Velasco-Forero S., Angulo J. Fully Trainable Gaussian Derivative Convolutional Layer. Proceedings of the 2022 IEEE International Conference on Image Processing (ICIP).

[44] Wei H., Wang Z., Hua G. Dynamically Mixed Group Convolution to Lighten Convolution Operation. Proceedings of the 2021 4th International Conference on Artificial Intelligence and Big Data, ICAIBD 2021.

[45] Chollet F. Xception: Deep Learning with Depthwise Separable Convolutions. Proceedings of the Proceedings—30th IEEE Conference on Computer Vision and Pattern Recognition, CVPR 2017.

[46] Paszke A., Gross S., Massa F., Lerer A., Bradbury Google J., Chanan G., Killeen T., Lin Z., Gimelshein N., Antiga L. PyTorch: An Imperative Style, High-Performance Deep Learning Library. Proceedings of the 33rd Annual Conference on Neural Information Processing Systems 2019, NeurIPS 2019.

[47] Yann L., Corinna C. (1998). Burges Christopher THE MNIST DATABASE of Handwritten Digits. Courant Inst. Math. Sci..

[48] Benavoli A., Corani G., Demšar J., Zaffalon M. (2017). Time for a Change: A Tutorial for Comparing Multiple Classifiers through Bayesian Analysis. J. Mach. Learn. Res..

[49] Corani G., Benavoli A., Demšar J., Mangili F., Zaffalon M. (2017). Statistical Comparison of Classifiers through Bayesian Hierarchical Modelling. Mach. Learn..

[50] Nilsson A., Smith S., Ulm G., Gustavsson E., Jirstrand M. A Performance Evaluation of Federated Learning Algorithms. Proceedings of the DIDL 2018—Proceedings of the 2nd Workshop on Distributed Infrastructures for Deep Learning, Part of Middleware 201.

[51] Crane M. (2018). Questionable Answers in Question Answering Research: Reproducibility and Variability of Published Results. Trans. Assoc. Comput. Linguist..

[52] Hintze J.L., Nelson R.D. (1998). Violin Plots: A Box Plot-Density Trace Synergism Statistical Computing and Graphics Violin Plots: A Box Plot-Density Trace Synergism. Source Am. Stat..

[53] Prechelt L. (2012). Early Stopping—But When?. Lect. Notes Comput. Sci..

[54] Choi D., Shallue C.J., Nado Z., Lee J., Maddison C.J., Dahl G.E. (2019). On Empirical Comparisons of Optimizers for Deep Learning. arXiv.

[55] Zeiler M.D. (2012). ADADELTA: An Adaptive Learning Rate Method. arXiv.

[56] Kingma D.P., Ba J.L. Adam: A Method for Stochastic Optimization. Proceedings of the 3rd International Conference on Learning Representations, ICLR 2015—Conference Track Proceedings.

[57] Loshchilov I., Hutter F. Decoupled Weight Decay Regularization. Proceedings of the 7th International Conference on Learning Representations, ICLR 2019.

[58] Luo L., Xiong Y., Liu Y., Sun X. Adaptive Gradient Methods with Dynamic Bound of Learning Rate. Proceedings of the 7th International Conference on Learning Representations, ICLR 2019.

[59] Dozat T. Incorporating Nesterov Momentum into Adam. Proceedings of the 4th International Conference on Learning Representations, ICLR 2016 —Conference Track Proceedings.

[60] Shao J., Hu K., Wang C., Xue X., Raj B. Is Normalization Indispensable for Training Deep Neural Networks?. Proceedings of the Advances in Neural Information Processing Systems.

[61] Huang L., Qin J., Zhou Y., Zhu F., Liu L., Shao L. (2020). Normalization Techniques in Training DNNs: Methodology, Analysis and Application. arXiv.

[62] Clanuwat T., Bober-Irizar M., Kitamoto A., Lamb A., Yamamoto K., Ha D. (1998). Deep Learning for Classical Japanese Literature. arXiv.

[63] Li L., Wang K., Li S., Feng X., Zhang L. (2020). LST-Net: Learning a Convolutional Neural Network with a Learnable Sparse Transform. Lect. Notes Comput. Sci..

